# HCSS-GB and IBESS: Secret Image Sharing Schemes with Enhanced Shadow Management and Visual-Gradient Access Control

**DOI:** 10.3390/e27090893

**Published:** 2025-08-23

**Authors:** Huanrong Pan, Wei Yan, Rui Wang, Yongqiang Yu

**Affiliations:** 1 College of Electronic Engineering, National University of Defense Technology, Hefei 230037, China; 2 Anhui Province Key Laboratory of Cyberspace Security Situation Awareness and Evaluation, Hefei 230037, China

**Keywords:** secret image sharing, Shamir secret sharing, Gaussian blur, bit expansion, efficient storage management, hierarchical access control

## Abstract

Image protection in privacy-sensitive domains, such as healthcare and military, exposes critical limitations in existing secret image sharing (SIS) schemes, including cumbersome shadow management, coarse-grained access control, and an inefficient storage-speed trade-off, which limits SIS in practical scenarios. Thus, this paper proposes two SIS schemes to address the above issues: the hierarchical control sharing scheme with Gaussian blur (HCSS-GB) and the image bit expansion-based sharing scheme (IBESS). For scenarios with limited storage space, HCSS-GB employs Gaussian blur to generate gradient-blurred cover images and integrates a controllable sharing model to produce meaningful shadow images without pixel expansion based on Shamir’s secret sharing. Furthermore, to accommodate real-time application scenarios, IBESS employs bit expansion to combine the high bits of generated shadow images with those of blurred carrier images, enhancing operational efficiency at the cost of increased storage overhead. Experimental results demonstrate that both schemes achieve lossless recovery (with PSNR of *∞*, MSE of 0, and SSIM of 1), validating their reliability. Specifically, HCSS-GB maintains a 1:1 storage ratio with the original image, making it highly suitable for storage-constrained environments; IBESS exhibits exceptional efficiency, with sharing time as low as 2.1 s under the (7,8) threshold, ideal for real-time tasks. Comparative analyses further show that using carrier images with high standard deviation contrast ($C_{\sigma }$) and Laplacian-based sharpness ($S_{L}$) significantly enhances shadow distinguishability, strengthening the effectiveness of hierarchical access control. Both schemes provide valuable solutions for secure image sharing and efficient shadow management, with their validity and practicality confirmed by experimental data.

## 1. Introduction

The rapid development of the digital economy has made data a critical national strategic resource and a fundamental driver of both economic and social progress. Images, due to universal accessibility and practical utility, have become an essential medium for transmitting information. Every day, billions of images are generated, stored, and shared, and the scale and influence of these images continue to expand. However, the widespread use of images has raised significant concerns regarding security and privacy [1]. Images often contain sensitive information, such as personal identities and location data. If such information is not properly protected during storage, transmission, or processing, the risk of privacy breaches increases. Furthermore, as image processing and analysis technologies continue to advance, the potential for unauthorized access, tampering, and misuse of images also rises. This situation poses threats not only to personal privacy and security but also to social stability and national security.

In multimedia security, traditional image protection focuses on three primary techniques: image encryption, which employs cryptographic algorithms such as 2D-enhanced logistic modular maps [2] and dynamic vector-level operations [3] to scramble or transform image content, preventing unauthorized access while balancing security and usability; and image steganography, which embeds secret data into carrier images through techniques like customized neighboring pixel differences [4] or compression-integrated methods [5], concealing the existence of secret information to achieve confidential transmission. However, both techniques share a critical vulnerability: single point of failure. To address this limitation, secret image sharing (SIS) has emerged as a robust alternative. SIS involves decomposing the original image into multiple shadow images using threshold mechanisms [6,7], enabling the reconstruction of the secret image from a subset of the distributed shadows and thus ensuring the confidentiality and integrity of the shared data.

Controlling user permissions is an important way to ensure the secure utilization of images, and the secure and efficient management of “ciphertext images” is a critical factor influencing the widespread application of image protection technologies. Thumbnail-preserving encryption (TPE) is a cryptographic paradigm that features capabilities in permission control and ciphertext management. Specifically, TPE balances image privacy and usability in cloud storage by preserving thumbnail features post-encryption, allowing authorized users to preview and recognize content while preventing unauthorized access to detailed visual information. Initially proposed by Wright et al. [8] in 2015 through a block-wise pixel shuffling method, TPE was vulnerable to statistical attacks due to unaltered pixel values. Subsequent TPE schemes have developed along two main lines: Ideal TPE and Approximate TPE. In Ideal TPE, the encrypted thumbnail maintains visual consistency with the original image and supports lossless decryption. Early work by Tajik et al. [9] introduced a substitution-permutation cipher framework, while Zhang et al. [10] leveraged chaotic systems to enhance security without sacrificing reversibility. Wen et al. [11] recently introduced TPE-DF, which employs dual-2D compressed sensing fusion with deterministic binary block diagonal (DBBD) and arbitrary scaling sensing degradation (ASSD) matrices to resist statistical attacks while preserving ideal, lossless decryption. These contributions collectively strengthen the Ideal TPE paradigm by improving both security and decryption reliability. Approximate TPE, which allows visual thumbnail approximations at the expense of lossless decryption, has evolved from early schemes like Marohn et al. [12]’s DRPE and TPE-LSB to later ones like HF-TPE [13] and PRA-TPE [14]. These improvements have enhanced both computational efficiency and the visual quality of shadow images. However, TPE’s need to preserve thumbnail visual features leaves it with potential visual information leakage.

Secret image sharing (SIS) originates from secret sharing (SS) technology. SS technology, since its foundation by Shamir [15] and Blakley [16] in the 1970s, has undergone significant evolution from theory to image applications. Shamir’s threshold scheme, based on polynomial interpolation, constructs a k−1 degree polynomial to generate share values for participants, achieving (k,n) threshold characteristics. Inspired by Shamir, Thien and Lin  [17] first applied Shamir’s polynomial-based SIS to digital images in 2002: they embedded secret pixels in all k−1 polynomial coefficients, sharing the secret image into shadow images sized 1/k of the original, laying the groundwork for image-based SS. Traditional SIS schemes often produced noisy shadow images, posing management and security challenges, leading researchers to develop meaningful secret image sharing (MSIS) [18], which generates comprehensible shadow images through two paradigms: information hiding fusion [19,20,21] and structure optimization [22,23,24]. The former embeds noisy shadows into natural cover images to enhance concealment, while the latter optimizes algorithms to make shadow pixel values approximate carrier pixel values, improving shadow image quality while ensuring reconstruction accuracy, thus significantly enhancing the understandability of shadow images and the security of SIS systems.

Recent studies on MSIS focus on enhancing security and share quality. Schemes employ hybrid fractal matrices [25], Boolean XOR [26], CRT [6], and saliency detection [18]. They generate meaningful shares indistinguishable from covers, support multi-secrets, and use operations like XOR or polynomial interpolation for recovery. Key advances include improved visual quality (via salient region optimization) and flexible parameters, ensuring both security and usability.

To enhance MSIS sharing efficiency, Yu et al. [27] integrated TPE with polynomial-based image secret sharing. Their scheme combines the visual accessibility of TPE with the security of polynomial secret sharing, improving image data confidentiality and integrity while boosting sharing speed. However, it still shares one secret image into multiple shadow images, complicating user management of cloud-stored images.

In this paper, we propose two solutions to address the issues such as cumbersome shadow management, coarse-grained access control, and an inefficient storage-speed trade-off: a hierarchical control sharing scheme with Gaussian blur (HCSS-GB), which is suitable for scenarios with high requirements on the attributes of shadow images; and an image bit expansion-based sharing scheme (IBESS), which features extremely high sharing efficiency and is suitable for scenarios with strict requirements on real-time performance. Hierarchical access control, as described herein, is a mechanism that differentiates user access to secret information based on the visual quality gradient of shadow images. Users with higher privileges receive shadow images with lower blur degrees, while users with lower privileges receive versions with higher blur degrees. The main contributions are summarized as follows:

(1) We have proposed two novel image sharing schemes that simultaneously achieve key characteristics including loss tolerance, simplified shadow image management, hierarchical access control, and resistance to privacy leakage—features that have not been concurrently realized in existing methods. This integration of multiple critical properties fills the gap in current research and provides new insights for secure image sharing and storage.
(2) The HCSS-GB adopts Gaussian blur to generate gradient-blurred carrier images and integrates a controlled sharing model based on Shamir’s secret sharing, generating shadow images without pixel expansion while maintaining the same format as the original plaintext image. This makes it suitable for scenarios requiring strict consistency between the attributes of shadow images and the plaintext image.
(3) The IBESS features high sharing efficiency by adopting a bit expansion mechanism, which sacrifices pixel format consistency but significantly accelerates the sharing process. This enables it to effectively meet the requirements of real-time processing in time-sensitive scenarios.

The structure of this paper is as follows: Section 1 introduces the background and significance of secret image sharing. Section 2 elaborates on the principles of Gaussian blur and the control sharing model. Section 3 clarifies the applicable scenarios of the proposed schemes. Section 4 presents the HCSS-GB with Gaussian blur and the fast preview algorithm. Section 5 details the IBESS. Section 6 outlines the experimental design and evaluation. Finally, Section 7 summarizes the work of this paper.

## 2. Preliminaries

This section establishes the theoretical foundations for the proposed schemes. We first delineate the principle of Gaussian blur. This technique is employed to generate a sequence of carrier images with distinct visual gradients, enabling the scheme’s hierarchical control based on visual quality. Subsequently, we introduce the control sharing model, which allows for the regulation of the visual effects of distributed shadow images, ensuring secure distribution of the secret image while enforcing threshold access control policies.

### 2.1. Gaussian Blur

Gaussian blur [28] serves as a pivotal technique in our schemes. The mathematical foundation is the Gaussian function defined as

(1) $$G\left(x,y\right)=\frac{1}{2\pi \sigma^{2}}e^{-\frac{x^{2}+y^{2}}{2\sigma^{2}}},$$

where *x* and *y* are spatial coordinate variables, σ represents the standard deviation that controls the blurring degree, and *e* denotes the natural logarithm base. The Gaussian blur operation is defined as the convolution of image function I(x,y) with the Gaussian kernel

(2) $$\left(I*G\right)\left(x,y\right)=\sum_{i=-\infty }^{\infty }\sum_{j=-\infty }^{\infty }I\left(x-i,y-j\right)\cdot G\left(i,j\right),$$

where I(x,y) represents input pixel values and (I∗G)(x,y) denotes the blurred output.

For digital implementation, a discrete convolution kernel of size (2n+1)×(2n+1) with $n\in Z^{+}$ is employed, where kernel elements are discretized by substituting spatial coordinates (x,y) in Equation (1) with discrete indices (i,j) for i,j∈[−n,n]; normalization ensures kernel elements sum to unity, such that the discrete Gaussian kernel G[i,j] constructed via this discretization is normalized as

(3) $$H\left[i,j\right]=\frac{G[i,j]}{\sum_{k=-n}^{n}\sum_{l=-n}^{n}G\left[k,l\right]},\;k,l\in \left[-n,n\right],$$

thereby ensuring accurate convolution. The blurred image B(x,y) is then computed as

(4) $$B\left(x,y\right)=\sum_{i=-n}^{n}\sum_{j=-n}^{n}I\left(x-i,y-j\right)\cdot H\left[i,j\right],$$

where I(x,y) denotes the input pixel value, H[i,j] represents the normalized kernel weights from Equation (3), and B(x,y) is the output blurred pixel value.

The standard deviation σ is a core parameter that directly determines the blurring intensity: a larger σ indicates a wider spread of the Gaussian distribution, leading to stronger blurring effects as more distant pixels contribute to the convolution result. In practical applications, σ typically ranges from 0.1 to 10. When σ approaches 0, the Gaussian kernel approximates a delta function, resulting in almost no blurring; when σ exceeds 10, the image tends to become overly blurred, losing most details.

The kernel size K×K, with *K* being an odd integer, determines the spatial range of pixel influence during blurring. A larger kernel size allows more surrounding pixels to participate in the convolution, which is necessary for achieving significant blurring effects, especially when σ is large. Common kernel sizes in practice include 3×3, 5×5, 7×7, up to 31×31 or larger for extreme blurring. However, excessively large kernels (e.g., K>31) may introduce computational redundancy without substantial improvement in blurring quality.

The relationship between σ and kernel size *K* is mutually constrained: a larger σ requires a larger kernel to capture the main energy of the Gaussian distribution, while an inappropriately small kernel for a given σ will truncate the distribution, causing inaccuracies in blurring. In practical applications, the relationship between kernel size K×K and standard deviation σ adheres to the Gaussian distribution truncation principle. The kernel size *K* is dynamically determined by σ via

(5) K=2⌈3σ⌉+1,

which ensures over 99.7% of the Gaussian distribution’s energy is encapsulated within the kernel, thereby preventing truncation artifacts. This formula balances blur quality with computational efficiency, as the complexity scales quadratically with *K*. For discrete implementations such as OpenCV, the constraint on σ can be derived as follows:

(6) $$\sigma \leq \frac{K-1}{6}.$$

This constraint ensures that the chosen σ is compatible with the kernel size *K*, avoiding energy loss due to insufficient kernel coverage. For example, a 5×5 kernel (K=5) constrains σ≤(5−1)/6≈0.67, while a 11×11 kernel allows σ≤(11−1)/6≈1.67, enabling stronger blurring with a larger kernel.

### 2.2. Control Sharing Model

This section focuses on the core algorithm and design concept of the control sharing model [27], which employs Shamir’s polynomial-based secret sharing scheme to share each pixel of the secret image *P*. The scheme operates as shown below.

For each secret pixel P(i,j), we generate shared values using the polynomial over the finite field GF(p):

(7) $$f\left(x\right)=\left(P\left(i,j\right)+\sum_{i=1}^{k-1}a_{i}x^{i}\right)\;\mathrm{mod}\;p\;,$$

where the shared pixel values are obtained by evaluating f(x) at different values of *x*. Here, the pixel values are represented as *b*-bit binary numbers. Specifically, for a pixel value in the range $\left[0,2^{b}-1\right]$, its *b*-bit binary representation $m_{0}m_{1}\ldots m_{b-1}$ corresponds to the decimal value $\sum_{i=0}^{b-1}m_{i}\times 2^{b-1-i}$. All operations are performed within the finite field GF(p), where *p* is a prime number chosen such that $p>2^{b}$ to ensure arithmetic correctness for image processing.

The values of f(x), which depend on the coefficients ${\left\{a_{i}\right\}}_{i=1}^{k-1}$, are iteratively generated through random selection until they satisfy the range specified by the following interval:

(8) $$f\left(x\right)\in \left[\sum_{g=1}^{\Omega (P,C)}m_{g-1}\times 2^{b-g},\;\sum_{g=1}^{\Omega (P,C)}m_{g-1}\times 2^{b-g}+2^{b-\Omega (P,C)}-1\right]\;,$$

where *P* and *C* represent corresponding pixels in the plaintext and cover images, respectively, and Ω(P,C) counts their number of consecutive identical most significant bits. Here, $m_{g}$ (g=0,1,…,b−1) denotes the *g*-th binary bit of the carrier image *C*’s pixel value, where $m_{0}$ is the most significant bit (MSB) and $m_{b-1}$ is the least significant bit (LSB). The interval in Equation (8) is constructed by fixing the first Ω(P,C) MSBs of *C*, which means that the lower bound of the interval is exactly the value represented by the first Ω(P,C) bits of *C* (with the remaining b−Ω(P,C) bits set to 0), and the upper bound is the maximum value when the remaining bits are set to 1. For example, if Ω(P,C)=3 and b=8, the interval is determined by ${\left(m_{0}m_{1}m_{2}000000\right)}_{2}$ to ${\left(m_{0}m_{1}m_{2}111111\right)}_{2}$, which ensures that f(x) shares the same $m_{0}m_{1}m_{2}$ pattern with *C*. By enforcing f(x) to lie within this interval, the shared values maintain the critical high-bit structure of *C*, enabling seamless embedding without altering the visual features encoded in the MSBs.

## 3. Application Scenario Settings

The proposed schemes (HCSS-GB and IBESS) are designed for storage-centric secret image sharing scenarios, where the core focus is on the efficient management, retrieval, and recovery of shadow images stored in systems (such as distributed cloud storage, local servers, or secure databases). The application scenario is set as follows.

- The dealer generates shadow images from the secret image through the sharing process and distributes them to authorized participants, who then store these shadow images in their respective storage systems. Shadow images are stored as files, and their organization is simplified by linking them to a single carrier image (via gradient Gaussian blur technology). This reduces redundancy, making it easier to index, categorize, and manage large sets of shadow images—particularly important for scenarios with multiple users or large-scale image libraries. When the secret needs to be reconstructed, authorized participants retrieve at least *k* stored shadow images.
- Within this storage-centric application scenario, shadow images are treated as integral files. In practice, stored shadow images typically exist in one of two states: they are either fully intact (retrievable and usable for recovery) or completely unavailable (e.g., due to storage failure or corruption, rendering them unfit for recovery). Therefore, robustness issues caused by noise during shadow image transmission are not considered.

Our schemes aim to adapt to this application scenario by optimizing the key stages covered by secret image sharing: generating shadow images that are easy to store and organize, implementing hierarchical access to simplify retrieval, and ensuring lossless recovery from completely retrieved shadow images.

## 4. Proposed HCSS-GB

This section presents a sharing scheme that synergistically combines the controlled sharing mechanism and Gaussian blur technique introduced in the preliminary knowledge section, where the controlled sharing mechanism ensures the secure generation and management of shadow images while the Gaussian blur technique plays a crucial role in enhancing their hierarchical management. By integrating these two methods, the proposed scheme offers enhanced flexibility and security, making it well-suited for diverse application scenarios. The relevant symbols are explained in Table 1. Later in this section, a Preview Sharing Phase is introduced to offer quick visual previews for parameter adjustment, guiding users in setting relevant parameters and enhancing scheme efficiency.

### 4.1. Overall Scheme Overview

**Figure 1:**
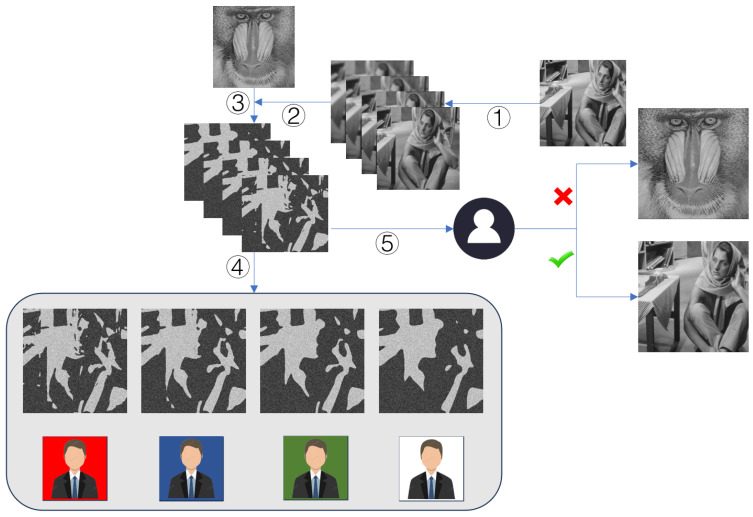
Flowchart of the HCSS-GB.

This section presents an overview of the HCSS-GB, detailing its operational framework and illustrating the sharing access control process through a flowchart. The scheme leverages gradient blur levels combined with permission tiers to enable fine-grained access control over secret images. The implementation process depicted in Figure 1 unfolds in the following sequential steps.

First, as shown in Step 1 of the figure, Gaussian blur is applied to the Barbara image to generate four Barbara images with progressively increasing blur levels, which serve as carrier images for the subsequent sharing process. Next, Steps 2–3 illustrate that the four blurred carrier images are input into the sharing system, where the control sharing model (introduced in the Preliminaries) employs Shamir’s polynomial-based scheme to share each pixel of the baboon secret image into four values. Specifically, the *i*-th share (i=1,2,3,4) is generated such that its most significant Ω bits match those of the *i*-th carrier image pixel, ensuring adherence to the high-bit structure of each corresponding carrier image. This process generates a shadow image set where the four shares are respectively associated with the four blurred Barbara images. In Step 4, during the access control phase, the system distributes shadow images with corresponding blur levels based on users’ pre-configured permission tiers: high-privilege users receive the least blurred images (retaining the most secret-related information), while lower-privilege users are allocated images with gradually increasing blur degrees. Finally, Step 5 demonstrates that unauthorized users, lacking the critical a priori knowledge, cannot correctly link shadow images to the secret image, thereby failing to access any meaningful information about the secret content. However, participants who possess knowledge of the correspondence between the carrier image and the secret image are capable of accurately reconstructing the secret image.

### 4.2. Gradient Gaussian Blurring of Carrier Image

To achieve hierarchical control and management of carrier images, this subsection introduces the gradient Gaussian blurring process for the carrier image *C*. The gradient blurring mechanism generates a series of blurred carrier images with varying degrees of blurriness, which is essential for the HCSS-GB.

The Gaussian blurring process is parameterized as follows:

- The initial standard deviation of the Gaussian blur is denoted as $\sigma_{0}$.
- The standard deviation increment is defined as $\Delta_{\sigma }$.
- The user’s sharing threshold is (k,n).
- The Gaussian blur kernel size *K* is dynamically determined by substituting $\sigma =\sigma_{0}+\Delta_{\sigma }\times \left(n-1\right)$ into Equation (5).

The detailed implementation steps for generating *n* blurred carrier images $BC_{1},BC_{2},\ldots$, $BC_{n}$ are described in Algorithm 1.
**Algorithm 1** Gradient Gaussian Blurring of Carrier Image**Input:** Carrier image *C*, initial standard deviation $\sigma_{0}$, standard deviation increment $\Delta_{\sigma }$, total number of shares *n*. **Output:** Blurred carrier images $BC_{1},BC_{2},\ldots ,BC_{n}$.
1:Compute $\sigma =\sigma_{0}+\left(n-1\right)\cdot \Delta_{\sigma }$2:Compute maximum kernel size *K* using Equation (5)3:Compute $BC_{1}$ as $\mathrm{GaussianBlur}(C,\sigma_{0},K)$4:**for** i=2 **to** *n* **do**5:   Set $\sigma_{i}=\sigma_{i-1}+\Delta_{\sigma }$6:   Compute $BC_{i}$ as $\mathrm{GaussianBlur}(C,\sigma_{i},K)$7:**end for**8:**return** $BC_{1},BC_{2},\ldots ,BC_{n}$


This gradient blurring process generates a sequence of carrier images by incrementally increasing the standard deviation σ. The resulting blurred carrier images $BC_{1},BC_{2},\ldots ,BC_{n}$ form the basis of the HCSS-GB, enabling fine-grained control over image sharing at varying blur levels.

### 4.3. Single-Pixel Sharing and Recovery

The following subsections detail the sharing and recovery operations performed at the pixel level for coordinates (i,j) using a (k,n)-threshold scheme, which serve as the fundamental building blocks of the HCSS-GB. Each pixel P(i,j) in the plaintext image *P* is treated as a secret value, and the sharing process leverages polynomial-based (k,n)-threshold secret sharing combined with the gradient Gaussian blurring technique.

#### 4.3.1. Sharing

For secret pixel $S=P\left(i,j\right)\in Z_{p}$ at coordinate (i,j) under a (k,n)-threshold scheme, a polynomial $P\left(x\right)=a_{0}+a_{1}x^{d_{1}}+\ldots +a_{k-1}x^{d_{k-1}}$ is generated as defined in Equation (7), where the secret *S* corresponds to the constant term $a_{0}$. The coefficient vector $a=(a_{0},a_{1},\ldots ,a_{k-1})$ includes the secret *S* and random coefficients $a_{1},\ldots ,a_{k-1}$ chosen from $Z_{p}$, ensuring the polynomial is of degree at most k−1.

The generalized Vandermonde matrix V for the (k,n)-threshold scheme at coordinate (i,j) is defined as

(9) $$V=\left[\begin{array}{cccc} x_{1}^{d_{0}} & x_{1}^{d_{1}} & \ldots & x_{1}^{d_{k-1}}\\ x_{2}^{d_{0}} & x_{2}^{d_{1}} & \ldots & x_{2}^{d_{k-1}}\\ \vdots & \vdots & \ddots & \vdots \\ x_{n}^{d_{0}} & x_{n}^{d_{1}} & \ldots & x_{n}^{d_{k-1}} \end{array}\right]\;\mathrm{mod}\;p,$$

where each $x_{i}$ is an element of the multiplicative group $Z_{p}^{\times }$, chosen as distinct non-zero integers modulo *p*. The exponents $d_{t}$ define the polynomial basis for the (k,n)-threshold secret sharing.

(k,n)-threshold shares for pixel P(i,j) are computed via the following linear transformation:

(10) $$s=Va^{⊤}\;\mathrm{mod}\;p,$$

where $a^{⊤}$ denotes the transpose of the coefficient vector. This transformation projects the secret and random coefficients onto the Vandermonde basis, generating *n* shares in the vector $s=(s_{1},s_{2},\ldots ,s_{n})$. Any *k* shares from s can reconstruct the coefficient vector a and thereby recover the secret *S*. The entire process of generating valid shares (including polynomial construction, Vandermonde matrix formation, and share computation) is formally outlined in Algorithm 2.

The shares must satisfy the following range constraint:

(11) $$s_{l}\in \left[\sum_{g=1}^{\Omega (P\left(i,j\right),BC_{l}\left(i,j\right))}m_{g-1}\times 2^{b-g},\sum_{g=1}^{\Omega (P\left(i,j\right),BC_{l}\left(i,j\right))}m_{g-1}\times 2^{b-g}+2^{b-\Omega (P\left(i,j\right),BC_{l}\left(i,j\right))}-1\right]$$

for l=1,2,…,n, where P(i,j) and $BC_{l}\left(i,j\right)$ denote pixel values at (i,j) in the plaintext and *l*-th blurred carrier image, respectively, and $\Omega (P\left(i,j\right),BC_{l}\left(i,j\right))$ counts consecutive identical MSBs for this coordinate in the (k,n)-threshold framework. Specifically, the validation of this range constraint is a critical step in Algorithm 2 (Step 5), where invalid shares trigger the regeneration of random coefficients to ensure share validity.

The notation $m_{0},m_{1},\ldots ,m_{b-1}$ represents the binary components of a pixel value in its *b*-bit representation, where $m_{0}$ denotes the most significant bit (MSB) and $m_{b-1}$ denotes the least significant bit (LSB). Each $m_{g}$ corresponds to the *g*-th bit position in the binary sequence.
**Algorithm 2** Single-Pixel Sharing Phase of the Proposed HCSS-GB**Input:** Pixel P(i,j) from the plaintext image *P*, threshold parameter *k*, finite field modulus *p*, blurred carrier images $BC_{1},\ldots ,BC_{n}$, exponents $d_{0},\ldots ,d_{k-1}$, coordinate (i,j). **Output:** Shared shares $s_{1},\ldots ,s_{n}$.
1:Set the polynomial constant term to the secret pixel: $a_{0}\leftarrow P\left(i,j\right)$, and randomly generate coefficients $a_{1},\ldots ,a_{k-1}\in Z_{p}$ for the polynomial defined in Equation (7).2:Select distinct non-zero elements $x_{1},\ldots ,x_{n}\in Z_{p}^{\times }$ and construct the generalized Vandermonde matrix V as specified in Equation (9).3:Form the coefficient vector $a=[a_{0},a_{1},\ldots ,a_{k-1}]$ and compute shares via the linear transformation in Equation (10).4:For each blurred carrier $BC_{l}$, determine the number of consecutive identical MSBs $\Omega (P\left(i,j\right),BC_{l}\left(i,j\right))$ at coordinate (i,j).5:Using the MSB count Ω, check if each share $s_{l}$ lies within the interval constraint defined in Equation (11).6:If any share $s_{l}$ fails the interval check, regenerate random coefficients $a_{1},\ldots ,a_{k-1}$ and repeat Steps 3–5 until all shares satisfy the interval constraint.7:Return the valid shares $s_{1},\ldots ,s_{n}$.


#### 4.3.2. Recovery

For qualified participants $X_{k}={\left\{x_{i}\right\}}_{i=1}^{k}$, the subsystem subsystem subsystem matrix $V_{k}$—a k×k submatrix of the generalized Vandermonde matrix V (Equation (9))—is derived by selecting rows of V corresponding to indices in $X_{k}$ while preserving the column structure defined by exponents $d_{0},d_{1},\ldots ,d_{k-1}$. This submatrix inherits the modular arithmetic properties of V, enabling secret pixel P(i,j) reconstruction via matrix inversion.

The recovery process begins with computing the determinant and its modular inverse, as formalized in Algorithm 3. The determinant is calculated as

(12) $$\Delta =\det(V_{k})\;\mathrm{mod}\;p,$$

and its inverse is derived using Fermat’s Little Theorem:

(13) $$\Delta^{-1}\equiv \Delta^{p-2}\;\mathrm{mod}\;p,$$

following the inversion principle outlined in the sharing section. Concurrently, the adjugate matrix is constructed through minor computations:

(14) $$\mathrm{adj}\left(V_{k}\right)={\left[{\left(-1\right)}^{i+j}M_{ji}\right]}_{i,j=1}^{k}\;\mathrm{mod}\;p,$$

where $M_{ji}$ denotes the determinant of the submatrix obtained by deleting the *j*-th row and *i*-th column of $V_{k}$, adhering to standard matrix theory.

The coefficient vector a is then reconstructed using the linear algebra inversion process, analogous to the sharing transformation in Equation (10):

(15) $$a=\Delta^{-1}\cdot \mathrm{adj}\left(V_{k}\right)s^{⊤}\;\mathrm{mod}\;p.$$

This step corresponds to the core reconstruction logic in Algorithm 3 (Step 4). From this vector, the secret *S* is recovered as the constant term $a_{0}$ (Algorithm 3, Step 5), corresponding to the polynomial defined in Equation (7). This approach efficiently leverages properties of finite fields and matrix algebra to ensure secure and accurate secret reconstruction.
**Algorithm 3** Single-Pixel Recovery Phase of the Proposed HCSS-GB**Input:** Shares $S_{k}={\left\{s_{i}\right\}}_{i=1}^{k}$, coordinate (i,j), subsystem matrix $V_{k}$, modulus *p*. **Output:** Recovered secret pixel P(i,j).
1:Compute the determinant Δ of the subsystem matrix $V_{k}$.2:Calculate the modular inverse $\Delta^{-1}$ using Fermat’s Little Theorem.3:Construct the adjugate matrix $\mathrm{adj}(V_{k})$ via cofactor expansion.4:Reconstruct the coefficient vector a by inverting $V_{k}$ using the formula $a=V_{k}^{-1}\cdot S_{k}$.5:Extract the secret pixel *S* from the constant term $a_{0}$ of the coefficient vector a.6:Map *S* to the pixel value at coordinate (i,j).


### 4.4. Preview Sharing Phase

To enhance operational efficiency and avoid unnecessary computational overhead caused by inappropriate parameter settings, this subsection introduces a fast preview mechanism. Unlike the HCSS-GB, its core design omits high-order bit matching operations, enabling rapid generation of preview shadow images. These images visually match the final shared results for Ω=1 and Ω=2, allowing users to validate parameter settings before performing full sharing. The detailed procedure is formalized in Algorithm 4.
**Algorithm 4** Preview Sharing Phase of the Proposed HCSS-GB**Input:** A plaintext image *P* and *n* blurred carrier images $BC_{1},BC_{2},\ldots ,BC_{n}$, all of size W×H and pixel bit-depth *b*; threshold parameters (k,n) where 2≤k≤n. **Output:** Preview shadow images $PSC_{1,1}$, $PSC_{1,2}$, …, $PSC_{1,n}$, $PSC_{2,1}$, $PSC_{2,2}$, …, $PSC_{2,n}$.
For each pixel position (w,h)∈{(w,h)|1≤w≤W,1≤h≤H}, perform Steps 2–3.Generate *n* secret shares $s_{1},s_{2},\ldots ,s_{n}$ using (k,n)-threshold secret sharing, where $s_{i}$ corresponds to the share for participant *i* and $s_{i}\in \left[0,2^{b}-1\right]$.For each participant i=1 to *n*:
$PSC_{1,i}\left(w,h\right)=\left(BC_{i}\left(w,h\right)\&2^{b-1}\right)|\left(s_{i}\&\left(2^{b-1}-1\right)\right)$, where & represents the binary AND operation, and | represents the bitwise OR operation;$PSC_{2,i}\left(w,h\right)=\left(BC_{i}\left(w,h\right)\&2^{b-2}\right)|\left(s_{i}\&\left(2^{b-2}-1\right)\right)$.
After processing all pixels, output the 2n preview shadow images $PSC_{1,1}$, $PSC_{1,2}$, …, $PSC_{1,n}$, $PSC_{2,1}$, $PSC_{2,2}$, …, $PSC_{2,n}$.


The algorithm processes each pixel in the plaintext image, generating n secret shares via (k,n)-threshold secret sharing. For preview construction, two types of shadow images are generated: $PSC_{1,i}$ retains the highest 1 bit of the carrier image’s pixel value while embedding shares in the lower b−1 bits; $PSC_{2,i}$ preserves the highest 2 bits with shares embedded in the lower b−2 bits.

This design ensures that previews for Ω=1 and Ω=2 visually approximate the final shared results without requiring high-order bit matching. The number of consecutive identical MSBs, Ω, dynamically adapts to the bit-depth b, maintaining generality across different image formats.

The preview mechanism enhances operational efficiency by generating visually representative previews for Ω=1 and 2. This allows users to validate (k,n) parameters and blur settings before full sharing, ensuring alignment with expectations while minimizing computational overhead.

### 4.5. Integrated Workflow of Hierarchical Control Sharing

This subsection presents the complete workflow of the HCSS-GB, which synergistically combines gradient Gaussian blurring with adaptive secret embedding strategies—this workflow is formally detailed in Algorithm 5. The algorithm adaptively selects optimal parameters based on preview feedback, enabling a balance between visual interpretability and cryptographic security. By leveraging the hierarchical structure of blurred carrier images, the scheme dynamically adjusts the number of significant bits Ω and Gaussian blur levels to meet diverse application requirements.
**Algorithm 5** Sharing Phase of the Proposed HCSS-GB**Input:** Plaintext image *P*, carrier image *C* (W×H); threshold (k,n) (2≤k≤n); initial standard deviation $\sigma_{0}$; deviation increment $\Delta_{\sigma }$. **Output:** *n* shadow images $SC_{1},SC_{2},\ldots ,SC_{n}$.
Generate *n* blurred carrier images $BC_{1},BC_{2},\ldots ,BC_{n}$ using Algorithm 1 with inputs *C*, $\sigma_{0}$, $\Delta_{\sigma }$, and *n*.Generate preview shadow images $PSC_{1,1},PSC_{1,2},\ldots ,PSC_{1,n},PSC_{2,1},PSC_{2,2},\ldots ,PSC_{2,n}$ using Algorithm 4 with inputs *P*, $BC_{1},BC_{2},\ldots ,BC_{n}$, and (k,n), then record $\left(\Omega ,\sigma_{l}\right)$ for all previews.Select the clearest preview $PSC_{\Omega_{2},x}$ and the most blurred preview $PSC_{\Omega_{1},y}$ such that $\Omega_{2}\geq \Omega_{1}$.Define $S_{x}=\left\{\sigma_{l}|\sigma_{l}\leq \sigma_{x}\right\}$ and $S_{y}=\left\{\sigma_{l}|\sigma_{l}\leq \sigma_{y}\right\}$, compute $m=\left\lceil \frac{x}{x+y}\cdot n\right\rceil$.If $|S_{x}|<m$, generate *m* sigmas in $S_{x}$ by creating an evenly spaced sequence of values between the minimum and maximum values in $S_{x}$.If $|S_{y}|<n-m$, generate n−m sigmas in $S_{y}$ by creating an evenly spaced sequence of values between the minimum and maximum values in $S_{y}$.Combine sigmas into $\Sigma =\left[S_{x}\left[m\right],S_{y}\left[n-m\right]\right]$, where $S_{x}\left[m\right]$ is constructed as m Gaussian blur standard deviations evenly distributed over the interval $\left[0,\sigma_{x}\right]$. Assign $\Omega_{i}=\left\{\begin{array}{cc} \Omega_{2}, & i\leq m\\ \Omega_{1}, & i>m \end{array}\right.$, sort pairs $\left(\Omega_{i},\Sigma_{i}\right)$ by Ω descending then σ ascending, and generate $SC_{i}$ using $\Sigma_{i}$ with MSB retention $\Omega_{i}$.Return $SC_{1},SC_{2},\ldots ,SC_{n}$ sorted by Ω and σ.


The Algorithm 5 achieves hierarchical control over sharing granularity by executing key operations defined in its steps. In Step 1, it generates a sequence of blurred carrier images $BC_{l}$ by varying the Gaussian blur parameter σ from $\sigma_{0}$ to $\sigma_{0}+\left(n-1\right)\Delta_{\sigma }$, establishing a blurring gradient for hierarchical sharing. Step 2 employs the preview mechanism to create 2n preview images for Ω=1 and 2, which supports parameter selection in Step 3—identifying the clearest preview (highest Ω) and most blurred preview (lowest Ω) to define the clarity range. Step 4 partitions σ values into sets $S_{x}$ and $S_{y}$ based on the selected previews, computing $m=\left\lceil \frac{x}{x+y}\cdot n\right\rceil$ to balance shares between clarity levels. In cases where $|S_{x}|<m$ or $|S_{y}|<n-m$, Steps 5 and 6 adaptively generate evenly spaced σ values within each set’s range to meet share count requirements. Finally, Step 7 assigns the higher clarity parameter $\Omega_{2}$ to the first *m* shares and $\Omega_{1}$ to the rest, sorting shares by Ω (descending) and σ (ascending) to produce a structured output. This workflow ensures adaptive control over share clarity while maintaining threshold security, even with imbalanced sigma distributions.

After presenting the sharing workflow, the recovery process for the HCSS-GB is formalized below. This algorithm integrates the recovery steps (Algorithm 6) as a core function while adding hierarchical parameter handling and share validation.
**Algorithm 6** Recovery Phase of the Proposed HCSS-GB**Input:** Authorized shadow images $SC_{x_{1}},SC_{x_{2}}\ldots ,SC_{x_{k}}$; authorized participant set $X_{k}=\left\{x_{1},x_{2},\ldots ,x_{k}\right\}$. **Output:** Recovered plaintext image *P*.
For each authorized share $SC_{x_{l}}$ (l=1..k), extract the pixel value $s_{l}$ at coordinate (i,j). Here, $s_{l}$ represents the shared pixel from the *l*-th authorized shadow image.Use the recovery process in Algorithm 3 with parameters $x_{1},x_{2},\ldots ,x_{k}$ (participant indices) and $s_{1},s_{2},\ldots ,s_{k}$ (extracted pixel values) to compute the original pixel P(i,j).Repeat Steps 1-2 for all coordinates (i,j) in the image domain to reconstruct the full plaintext image *P*.


## 5. Proposed IBESS

This section elaborates on the IBESS, a high-efficiency SIS scheme tailored for real-time scenarios. It first outlines the core design principles and overall framework of IBESS, emphasizing its bit expansion mechanism that enables rapid sharing. Subsequently, the specific processes of shadow image generation and secret image reconstruction, along with the underlying algorithms, are detailed to fully demonstrate how IBESS achieves lossless recovery while balancing speed and storage trade-offs.

### 5.1. Scheme Overview

This scheme achieves efficient sharing and lossless recovery by embedding secret shares into image pixels with an increased bit depth from 8 bits to 16 bits. It is suitable for applications where fast sharing is prioritized and storage space is not a critical concern.

Each shadow pixel is constructed by combining the high bits of the carrier image, random values, and the secret share:

(16) $$S_{l}\left(i,j\right)=\left(\left(\left\lfloor \frac{BC_{l}\left(i,j\right)}{2^{8-\Omega }}\right\rfloor \ll \left(8-\Omega \right)\right)|R_{8-\Omega }\right)\ll 8\;|\;{\mathrm{share}}_{l}\left(i,j\right),$$

where Ω∈[1,8] controls the trade-off between visual similarity and sharing security, $BC_{l}\left(i,j\right)$ represents the pixel value of the carrier image after Gaussian blur, ${\mathrm{share}}_{l}\left(i,j\right)$ is the secret share, and $R_{8-\Omega }$ denotes a (8−Ω)-bit random integer.

For each pixel (i,j), the secret shares are generated by evaluating the polynomial defined in Equation (7) at l=1,2,…,n:

(17) $${\mathrm{share}}_{l}\left(i,j\right)=f\left(l\right),$$

where each f(l) represents a share of the pixel value P(i,j) generated by the Shamir secret sharing scheme.

### 5.2. Sharing Process

The sharing process involves generating blurred carrier images, sharing each pixel of the secret image using a threshold scheme, and constructing shadow images by combining the high bits of the blurred carrier images with the secret shares. The pseudocode for the sharing process is provided in Algorithm 7.
**Algorithm 7** Sharing Phase of the Proposed IBESS**Input:** A secret image *P* and a carrier image *C*, both of size W×H; threshold parameters (k,n), where 2≤k≤n; initial standard deviation $\sigma_{0}$; deviation increment $\Delta_{\sigma }$. **Output:** The *n* shadow images $SC_{1},SC_{2},\ldots ,SC_{n}$.
Generate *n* blurred carrier images $BC_{1},BC_{2},\ldots ,BC_{n}$ with standard deviations $\sigma_{l}=\sigma_{0}+\left(l-1\right)\Delta_{\sigma }$ for l=1,2,…,n.For each pixel (i,j) in *P*:
Share P(i,j) using the polynomial defined in Equation (7) to generate *n* shares ${\mathrm{share}}_{l}\left(i,j\right)=f\left(l\right)$ for l=1,2,…,n.Construct shadow pixel $SC_{l}\left(i,j\right)$ by combining high bits of $BC_{l}$ and the secret share as defined in Equation (16).
Output the *n* shadow images $SC_{1},SC_{2},\ldots ,SC_{n}$.


### 5.3. Recovery Process

The recovery process involves reconstructing the secret image from *k* shadow images using Lagrange interpolation, as defined by the Shamir secret sharing scheme. The pseudocode for the recovery process is provided in Algorithm 8, with the Lagrange formula explicitly included for clarity.
**Algorithm 8** Recovery Phase of the Proposed IBESS**Input:** *k* shadow images $SC_{1},SC_{2},\ldots ,SC_{k}$ and their corresponding positions 1,2,…,k; modulus *p*. **Output:** The reconstructed secret image $P^{\prime }$.
Initialize an empty image $P^{\prime }$ of size W×H.For each pixel position (i,j) in $P^{\prime }$:
Extract the low 8 bits from each $SC_{m}\left(i,j\right)$ to obtain secret shares $s_{m}$ for m=1,2,…,k.Compute the reconstructed pixel $P^{\prime }\left(i,j\right)$ using Lagrange interpolation:$$P^{\prime }\left(i,j\right)=\sum_{m=1}^{k}s_{m}\cdot \prod_{\begin{array}{c} t=1\\ t\neq m \end{array}}^{k}\frac{0-t}{m-t}\;\mathrm{mod}\;257.$$
Output the reconstructed secret image $P^{\prime }$.


The algorithm processes each pixel position by extracting secret shares from the low 8 bits of the shadow images, then applies Lagrange interpolation to reconstruct the original pixel values. This scheme achieves lossless recovery, efficient sharing, and hierarchical control over sharing granularity, making it suitable for applications prioritizing both security and real-time performance with flexible storage considerations.

## 6. Experimental Design and Evaluation

In this section, we first demonstrate the sharing effects of the two schemes. Our evaluation then focuses on measuring the SSIM and PSNR values between the generated shadow images and cover images, which serves a dual purpose: verifying the hierarchical controlled sharing mechanism of the shadow images and providing practical suggestions for selecting appropriate cover images. Additionally, to verify the feasibility of adjusting the Gaussian blur parameters (initial standard deviation $\sigma_{0}$ and standard deviation increment $\Delta_{\sigma }$) through the preview sharing phase of HCSS-GB, we evaluate the SSIM and PSNR values between the preview shadow images (PSC) and the shadow images (SC). Then, we assess the performance of the schemes by evaluating two key metrics: the sharing time required for each scheme and the storage space occupied by the generated shadow images. Finally, we compare with related schemes to discuss the advantages of our two proposed schemes.

**Figure 2:**
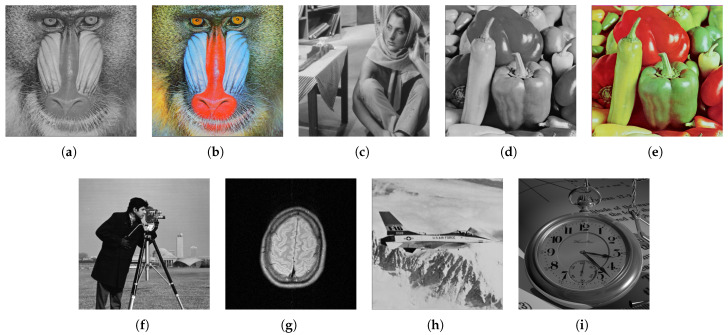
The experimental images used in this section: (**a**) baboon; (**b**) baboonRGB; (**c**) barbara; (**d**) peppers; (**e**) peppersRGB; (**f**) cameraman; (**g**) brain; (**h**) plane; (**i**) watch.

The experimental setup utilizes an Intel Core i5-12450H CPU with 16 GB RAM, operating on Windows 11 OS. The software environment consists of Python 3.9, OpenCV 4.11.0.86, and NumPy 2.0.2. We employed a dataset comprising nine 8-bit images for our experiments, including two 512 × 512 RGB images and seven 256 × 256 grayscale images as shown in Figure 2. These images encompass a range of textures from dense to sparse and intricate to simple structural outlines, providing a robust basis for evaluating the performance of our sharing schemes across diverse image complexity scenarios.

### 6.1. Property Illustration

This subsection presents experimental results to demonstrate the fundamental functionalities of the two proposed schemes (HCSS-GB and IBESS). Through visual validation of shadow image generation, secret recovery, and difference analysis, we verify the core properties of both schemes, including lossless reconstruction, generation of shadow images without pixel expansion, and resistance to privacy leakage, thereby confirming their practical feasibility.

**Figure 3:**
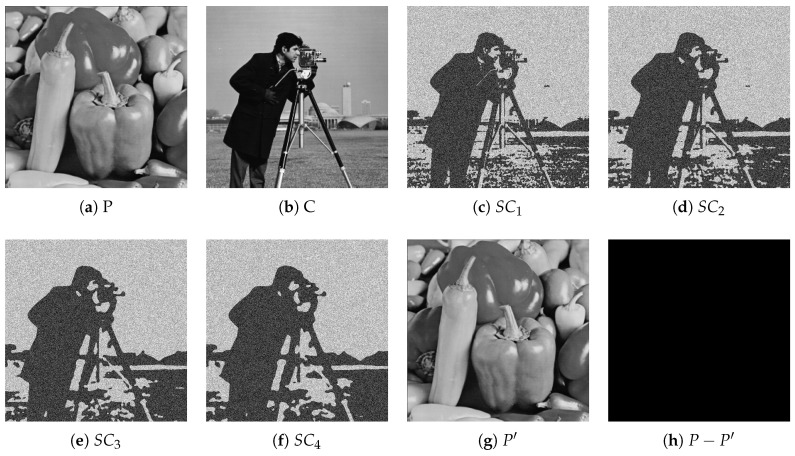
Experimental results of HCSS-GB with (2,4) threshold secret sharing scheme and Gaussian blur parameters $\sigma_{0}=1.0$, $\Delta_{\sigma }=1.0$: (**a**) original plaintext image *P* (peppers); (**b**) carrier image *C* (cameraman); (**c**–**f**) shadow images $SC_{1}$–$SC_{4}$; (**g**) recovered image $P^{\prime }$; (**h**) difference image $P-P^{\prime }$.

Figure 3 illustrates the experimental results of HCSS-GB under the (2, 4) threshold with Gaussian blur parameters $\sigma_{0}=1.0$ and $\Delta_{\sigma }=1.0$. It shows the original plaintext image *P* (peppers), carrier image *C* (cameraman), four generated shadow images $SC_{1}$-$SC_{4}$, the recovered image $P^{\prime }$, and the difference image $P-P^{\prime }$. This figure demonstrates that HCSS-GB achieves lossless recovery (evidenced by the all-zero difference image), generates shadow images without pixel expansion (consistent with the original resolution), and ensures shadow images retain the visual features of the carrier image while being unrelated to the plaintext, thus verifying its shadow management and privacy protection capabilities.

**Figure 4:**
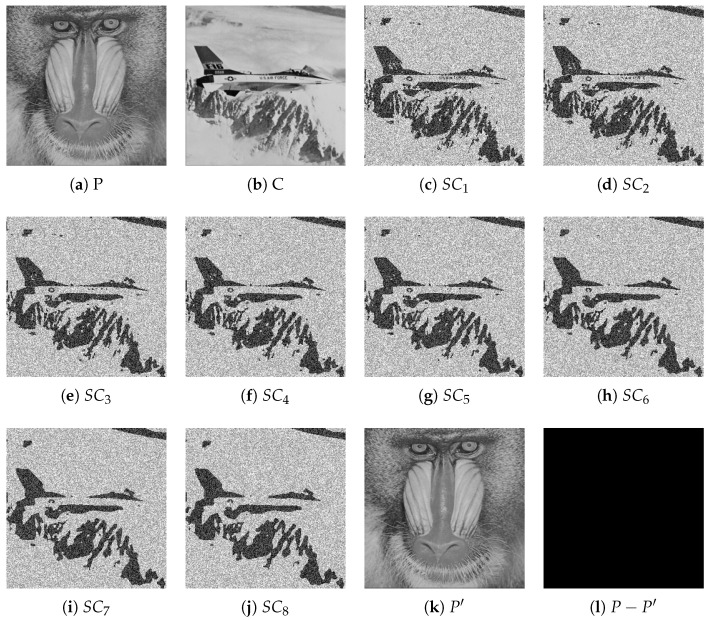
Experimental results of IBESS with (4, 8) threshold secret sharing scheme and Gaussian blur parameters $\sigma_{0}=0.3$, $\Delta_{\sigma }=0.2$: (**a**) original plaintext image *P* (baboon); (**b**) carrier image *C* (plane); (**c**–**j**) shadow images $SC_{1}$–$SC_{8}$; (**k**) recovered image $P^{\prime }$; (**l**) difference image $P-P^{\prime }$.

Figure 4 presents the results of IBESS under the (4, 8) threshold with $\sigma_{0}=0.3$ and $\Delta_{\sigma }=0.2$, including the original image *P* (baboon), carrier image *C* (plane), eight shadow images $SC_{1}$-$SC_{8}$, the recovered image $P^{\prime }$, and the difference image. It validates that IBESS maintains lossless recovery (with $P^{\prime }\equiv P$) despite using bit expansion, and its generated shadow images, while having 16-bit depth, still preserve the structural characteristics of the blurred carrier, confirming its high efficiency and reliability.

**Figure 5:**
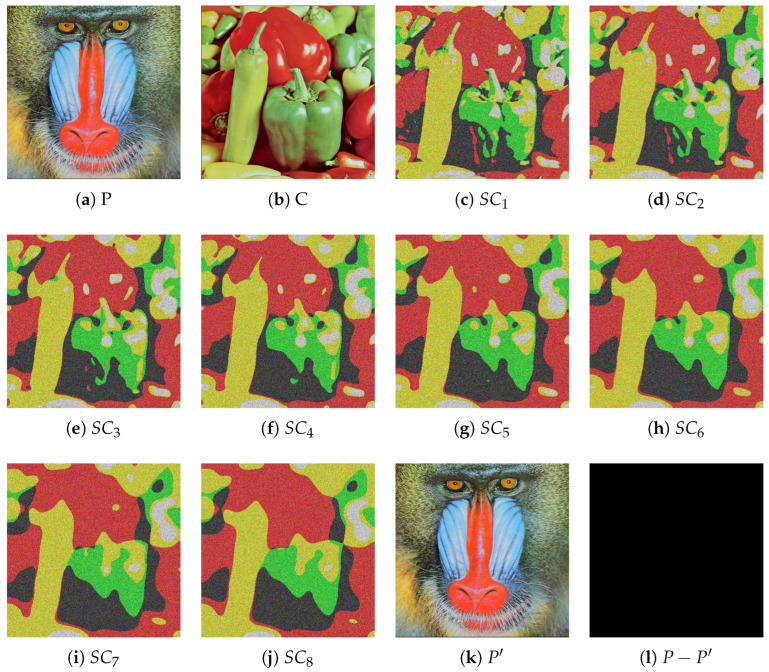
Experimental results of HCSS-GB with (5,8) threshold secret sharing scheme and Gaussian blur parameters $\sigma_{0}=2.0$, $\Delta_{\sigma }=2.0$: (**a**) original plaintext image *P* (baboonRGB); (**b**) carrier image *C* (peppersRGB); (**c**–**j**) shadow images $SC_{1}$–$SC_{8}$; (**k**) recovered image $P^{\prime }$; (**l**) difference image $P-P^{\prime }$.

Figure 5 further shows HCSS-GB’s performance on RGB images under the (5, 8) threshold with $\sigma_{0}=2.0$ and $\Delta_{\sigma }=2.0$, using *P* (baboonRGB) and *C* (peppersRGB). The results indicate that HCSS-GB is applicable to color images, maintaining consistent properties of lossless recovery and non-expanded shadow resolution, thus demonstrating its versatility across image types.

### 6.2. Visual Quality Comparison

In this section, we conduct a comprehensive evaluation of the visual quality between the shadow images generated by the proposed schemes and the corresponding carrier images, as well as between the reconstructed images and the original secret images, in order to validate the effectiveness of the proposed schemes. Furthermore, we assess the visual fidelity of both shadow and carrier images produced by the phase of preview sharing in the HCSS-GB, thereby demonstrating the enhancements of effectiveness. Finally, we perform an analysis of the texture and luminance characteristics of the carrier images to investigate potential criteria for selecting suitable carrier images.

#### 6.2.1. Quality Evaluation Metrics

We first present a detailed description of the visual quality evaluation metrics employed in the experiment. SSIM and PSNR [29] are recognized as critical quantitative indicators for evaluating image fidelity, and are utilized to measure the degree of similarity between two images. Standard deviation contrast ($C_{\sigma }$) and Laplacian-based sharpness ($S_{L}$) are applied to characterize the inherent visual features of carrier images.

For evaluating similarity between carrier and shadow images, SSIM is employed as a perceptual metric. It considers luminance, contrast, and structural information, defined by the following:

(18) $$\mathrm{SSIM}\left(x,y\right)=\frac{\left(2\mu_{x}\mu_{y}+c_{1}\right)\left(2\sigma_{x,y}+c_{2}\right)}{\left(\mu_{x}^{2}+\mu_{y}^{2}+c_{1}\right)\left(\sigma_{x}^{2}+\sigma_{y}^{2}+c_{2}\right)},$$

where $\mu_{x}$ and $\mu_{y}$ are luminance means, $\sigma_{x}^{2}$, $\sigma_{y}^{2}$ are variances, and $\sigma_{x,y}$ is the covariance. SSIM values closer to 1 indicate higher similarity, aligning with human visual perception. By maintaining consistent parameters across different carrier images, this study establishes a basis for rational carrier image selection.

Similarly, PSNR is utilized to quantify the peak error between the carrier image and the shadow image. It is defined as follows:

(19) $$\mathrm{PSNR}=10\log_{10}\left(\frac{{\mathrm{MAX}}^{2}}{\mathrm{MSE}}\right),$$

where MAX is the maximum possible pixel value of the image (for 8-bit images, MAX = 255), and MSE [30] is the mean squared error calculated as follows:

(20) $$\mathrm{MSE}=\frac{1}{WH}\sum_{i=1}^{W}\sum_{j=1}^{H}{\left[C\left(i,j\right)-SC\left(i,j\right)\right]}^{2},$$

with *W* and *H* being the width and height of the image, respectively. As a commonly used metric for assessing image quality, higher PSNR values indicate better quality. Notably, when comparing an image to itself, the PSNR theoretically approaches infinity due to the absence of differences. By incorporating both SSIM and PSNR, this study comprehensively evaluates the similarity between carrier and shadow images.

To assess the textural and structural characteristics of images, we employs two quantitative indicators, $C_{\sigma }$ and $S_{L}$. $C_{\sigma }$ reflects the degree of pixel intensity variation within the image. A higher value indicates a greater spread of pixel values, implying stronger contrast and more distinct visual features in an image. $C_{\sigma }$ is calculated as follows:

(21) $$C_{\sigma }=\sqrt{\frac{1}{WH}\sum_{i=1}^{W}\sum_{j=1}^{H}{\left(C\left(i,j\right)-\mu_{C}\right)}^{2}},$$

where C(i,j) represents the pixel intensity at position (i,j) in the image. *W* and *H* denote the width and height of the image, respectively. $\mu_{C}$ is the mean pixel value of the image, defined as:

(22) $$\mu_{C}=\frac{1}{WH}\sum_{i=1}^{W}\sum_{j=1}^{H}C\left(i,j\right).$$

$S_{L}$ quantifies the magnitude of high-frequency components in an image, which correspond to edges and fine details. A higher value indicates sharper image features and more pronounced edges. $S_{L}$ is calculated by first applying the Laplacian operator to the image and then computing the variance of the resulting response, which can be expressed as follows:

(23) $$S_{L}=\sum_{i=1}^{W}\sum_{j=1}^{H}{\left(L\left(i,j\right)-\mu_{L}\right)}^{2},$$

where L(i,j) represents the Laplacian response at position (i,j), and $\mu_{L}$ is the mean of the Laplacian response across the entire image.

#### 6.2.2. Visual Quality of Shadow Images

The visual quality comparison between the shadow images and the carrier images is a crucial step in verifying the effectiveness of the two schemes proposed in this paper. In this section, we use SSIM and PSNR to compare the different shadow images generated by different schemes with their corresponding carrier images.

**Figure 6:**
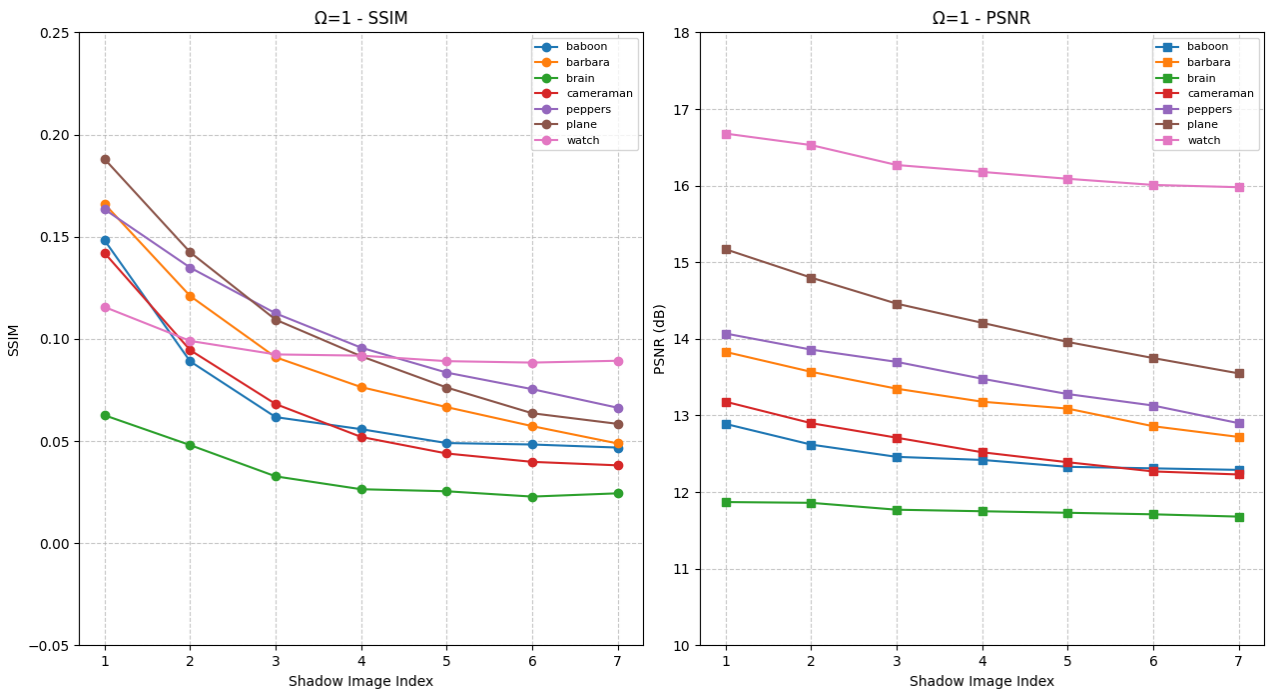
SSIM and PSNR curves for evaluating the fidelity between shadow images $SC_{i}$ and the original carrier image *C* under different Gaussian blur levels for HCSS-GB with Ω=1, initial standard deviation $\sigma_{0}=1$ and increment $\Delta_{\sigma }=1$.

First, the effectiveness of the proposed HCSS-GB scheme is validated. The HCSS-GB scheme initially applies Gaussian blurring to the carrier image with varying levels of intensity. Then, it employs a polynomial-based SS and controllable sharing model to share the secret image. This process generates shadow images that are, to varying degrees, similar to the carrier image, thereby enabling user access control and efficient management of the shadow images. Figure 6 presents the SSIM and PSNR values of shadow images generated by the HCSS-GB scheme after processing various experimental images, in comparison with the corresponding unblurred carrier images under the condition Ω=1. The horizontal axis denotes the various shadow images, while the vertical axis indicates the corresponding evaluation results. Each curve represents a distinct experimental image. Specifically, the differences among shadow images arise from carrier images that have been processed using varying Gaussian blur parameters σ. As the value of σ increases, the degree of blurring in $BC_{i}$ (the processed carrier image) also increases, consequently affecting the similarity between $SC_{i}$ (the shadow image) and the original carrier image *C*. This is reflected in the gradual decrease of both SSIM and PSNR values. The shadow images retain the structural information of the original carrier image, as well as variations in the visual appearance of different shadow images.

**Figure 7:**
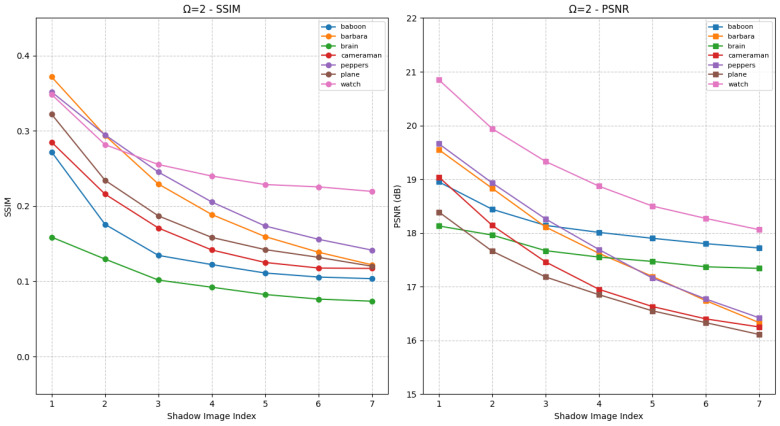
SSIM and PSNR curves for evaluating the fidelity between shadow images $SC_{i}$ and the original carrier image *C* for HCSS-GB with Ω=2, initial standard deviation $\sigma_{0}=1$ and increment $\Delta_{\sigma }=1$.

Figure 7 presents the experimental results under the condition Ω=2, in comparison with Figure 6. In this case, the two most significant bits of each pixel in the shadow image are identical to those of the corresponding blurred carrier image. The visual quality of the shadow images is notably enhanced, as increased SSIM and PSNR values relative to Figure 6. Furthermore, as parameters vary, distinct differences in visual quality among the shadow images become apparent, reflected in substantial fluctuations in SSIM and PSNR. Users with higher privileges are granted access to shadow images with enhanced visual fidelity, whereas those with lower privileges receive images of reduced quality. Therefore, as demonstrated in Figure 6 and Figure 7, the proposed HCSS-GB scheme supports differentiated access control across distinct user levels.

Then, we evaluate the visual quality of the shadow images generated by the proposed IBESS scheme. For the IBESS scheme, we first perform Gaussian blurring on the carrier image, followed by polynomial-based SS and bit-expansion to generate shadow images $SC_{i}$. This workflow ensures $SC_{i}$ retains similarity to *C* while enabling access control.

**Figure 8:**
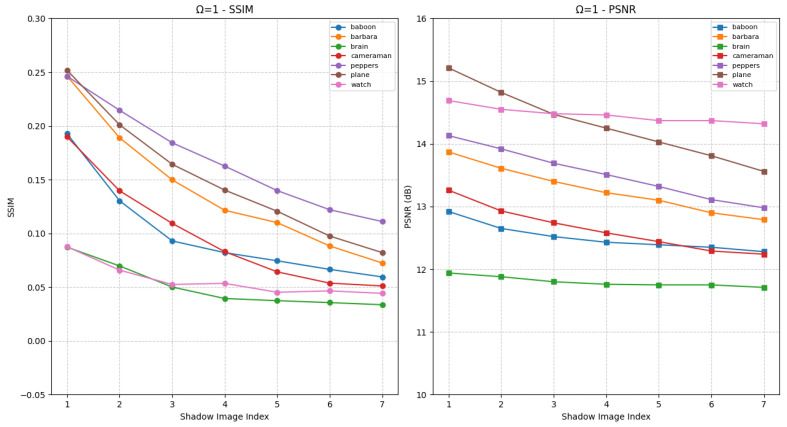
SSIM and PSNR curves for evaluating the fidelity between shadow images $SC_{i}$ and the original carrier image *C* under different Gaussian blur levels for IBESS with Ω=1, initial standard deviation $\sigma_{0}=1$ and increment $\Delta_{\sigma }=1$.

Figure 8 shows IBESS results under Ω=1. The horizontal axis represents shadow images derived from carrier images with different σ, and the vertical axis shows SSIM/PSNR. As σ increases, $BC_{i}$ becomes more distorted, reducing $SC_{i}$-to-*C* similarity. Despite this, $SC_{i}$ preserves core structural features of *C*, with visual differences across varying σ.

**Figure 9:**
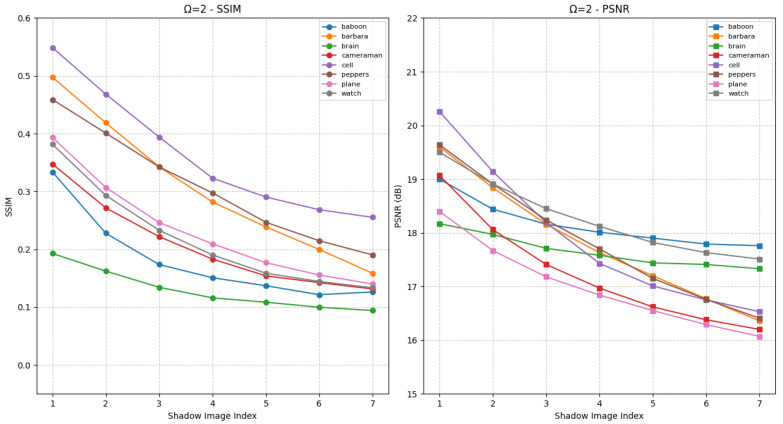
SSIM and PSNR curves for evaluating the fidelity between shadow images $SC_{i}$ and the original carrier image *C* under different Gaussian blur levels for IBESS with Ω=2, initial standard deviation $\sigma_{0}=1$ and increment $\Delta_{\sigma }=1$.

Under Ω=2, as shown in Figure 9, the two most significant bits of $SC_{i}$ pixels match the blurred carrier. This matching mechanism leads to higher SSIM and PSNR values compared to Ω=1, effectively improving the visual fidelity of shadow images. When σ varies, noticeable fluctuations in SSIM and PSNR emerge, which directly enable privilege-based differentiation: high-privilege users can access $SC_{i}$ with higher fidelity, while low-privilege users receive lower-quality versions.

Similar to HCSS-GB, IBESS leverages Gaussian blur and sharing mechanisms to support hierarchical access control. Through SSIM/PSNR analysis, we confirm IBESS balances visual fidelity and access differentiation, ensuring $SC_{i}$ retains recognizable structural information while aligning with user privilege levels.

#### 6.2.3. Visual Quality of the Recovered Image

The evaluation of the visual quality of the recovered image serves to validate the performance of the two proposed schemes in both sharing and recovering the secret image. Lossless recovery refers to the exact equivalence between the secret image and the recovered image, with no information loss. This property holds significant practical value across various application domains. In this section, we use three visual quality indicators, namely PSNR, MSE and SSIM, to conduct experiments on different threshold parameters and different secret images to verify the differences between the recovered images generated by the proposed two schemes and the original secret images. Experimental results are summarized in Table 2. The achieved PSNR of *∞*, MSE of 0, and SSIM of 1 collectively demonstrate that both HCSS-GB and IBESS successfully realize lossless recovery of the secret image.

#### 6.2.4. Selection of Carrier Images

In this section, we utilize metrics $C_{\sigma }$ and $S_{L}$ to assess the fundamental characteristics of the carrier image. By integrating the PSNR and SSIM values of the generated shadow images, we investigate the optimal carrier image that enables the proposed schemes to fully demonstrate its advantages.

First, we calculated the values of $C_{\sigma }$ and $S_{L}$ for the experimental images, and the results are shown in Table 3. It can be seen that all experimental images share consistent trends in SSIM and PSNR changes under Gaussian blur variations, regardless of the scheme (HCSS-GB or IBESS) or Ω value (1 or 2). As the Gaussian blur standard deviation increases, both SSIM and PSNR values of shadow images generally show a decreasing trend, indicating reduced fidelity between shadow images $SC_{i}$ and the original carrier image *C*. This universal pattern reflects the inherent impact of Gaussian blur on image structural similarity and pixel intensity consistency.

For images with higher $C_{\sigma }$ values (e.g., cameraman with $C_{\sigma }=62.34$), the decline in SSIM and PSNR is more pronounced with increasing blur, meaning they are more sensitive to Gaussian blur. In contrast, images with lower $C_{\sigma }$ values (e.g., baboon with $C_{\sigma }=32.71$) exhibit a relatively gentle reduction in SSIM and PSNR, showing stronger resistance to blur effects. Regarding $S_{L}$, images with greater structural complexity (e.g., cameraman with $S_{L}=2241.4243$) tend to maintain higher initial SSIM and PSNR under small blur levels but also face steeper quality degradation as blur intensifies.

Based on these universal trends, we recommend selecting carrier images according to specific application requirements. For scenarios demanding strict access control with distinct quality differentiation between privilege levels, images with high $C_{\sigma }$ (such as cameraman) are preferable, as small changes in blur can create noticeable quality gaps. When a balance between access control granularity and stable shadow image recognizability is needed, images with moderate $C_{\sigma }$ and $S_{L}$ (e.g., barbara or plane) are suitable. For applications where shadow image quality must remain relatively consistent across different privilege levels despite blur variations, images with low $C_{\sigma }$ (like baboon) should be chosen, leveraging their blur-resistant properties to ensure basic usability for all user tiers.

### 6.3. Performance Comparison

In real-world applications, the sharing efficiency of image security schemes is a critical factor, especially for scenarios requiring real-time processing or batch operations. This section evaluates the sharing time performance of the proposed HCSS-GB and IBESS. By analyzing the computational overhead under different threshold settings, we aim to provide insights into the practical applicability and efficiency trade-offs of the two schemes. The sharing time was measured for various threshold configurations to reflect the impact of security requirements on computational efficiency.

**Figure 10:**
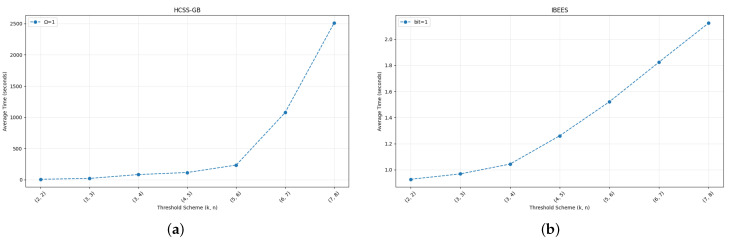
Sharing time comparison between HCSS-GB and IBESS under varying threshold parameters. (**a**) Sharing time of HCSS-GB; (**b**) Sharing time of IBESS.

Figure 10a depicts the average sharing time for different threshold parameters of HCSS-GB. As the threshold value increases, the sharing time shows a significant upward trend. Specifically, the (2,2)-threshold HCSS-GB requires the least time, approximately 10 s, while the (7,8)-threshold demands the most time, reaching about 2500 s. This trend is primarily attributed to the iterative screening process inherent in the controlled sharing mechanism, which becomes increasingly computationally intensive with higher threshold values.

In the sharing process, the core time-consuming steps lie in the controlled sharing logic specified in Steps 4–6 of Algorithm 2. For each blurred carrier image $BC_{l}$, the algorithm first determines the number of consecutive identical MSBs $\Omega (P\left(i,j\right),BC_{l}\left(i,j\right))$, then checks if each share $s_{l}$ satisfies the interval constraint derived from Ω. If any share fails this check, the algorithm must regenerate random coefficients $a_{1},\ldots ,a_{k-1}$ and repeat the share generation and validation process until all shares meet the constraint. As the threshold *k* increases, the number of blurred carriers $BC_{l}$ and corresponding shares $s_{l}$ to check grows, amplifying the complexity of the MSB matching and interval validation. Moreover, higher *k* raises the likelihood of share failures due to stricter constraints, leading to more frequent regeneration cycles. This iterative screening, unique to the controlled sharing model, drives the significant increase in sharing time.

Figure 10b illustrates the average sharing time for different threshold schemes in the IBESS. Unlike the control sharing model, the increase in sharing time with threshold values is relatively moderate. The (2,2)-threshold IBESS scheme takes only 0.7 s, and the (7,8)-threshold scheme requires approximately 2.1 s. This suggests that the IBESS is more efficient in handling higher threshold values compared to the HCSS-GB.

The efficiency of IBESS stems from its streamlined share generation process, which avoids the iterative screening steps inherent in controlled sharing. Unlike the control model, IBESS does not require repeated checks of MSB matching (Ω) or interval constraints for shares, nor does it involve regenerating polynomial coefficients due to failed validations. Instead, it directly constructs polynomials and generates shares in a single pass, eliminating the cumulative overhead of iterative screening. This optimized workflow allows IBESS to maintain efficiency even as threshold values increase, resulting in the relatively gentle upward trend observed in the sharing time curve.

Table 4 lists the ratio of storage space between the original image and the per-shadow image generated by our proposed schemes. Both schemes produce shadows at the same resolution and dimensions, so differences stem solely from pixel bit-depth and compression. IBESS expands each 8-bit pixel to 16 bits; lossless compression brings the footprint to slightly less than 2× (≈ 2×). HCSS-GB retains the native 8-bit format without compression, yielding an exact 1× ratio. Hence, IBESS trades approximately doubled storage for speed, whereas HCSS-GB preserves size parity at the expense of slower sharing, with performance also affected by computer configurations.

### 6.4. Key Scheme Characteristics Comparison

To further clarify the advantages of the proposed schemes, we present a detailed comparison of key characteristics in Table 5, covering five critical dimensions. The evaluation is based on the core mechanisms and experimental results of each scheme, with specific analysis as follows:

- **Loss Tolerance:** This feature refers to the ability to recover the original secret image when up to n−k shadow images are lost or damaged, which is critical for ensuring data integrity in distributed storage. Cheng et al.’s scheme [24] is based on polynomial (k,n) threshold secret sharing, where *k* or more shadows can reconstruct the secret, thus supporting loss tolerance. Chowdhury et al.’s TPE scheme [31] generates a single shadow image, lacking multi-shadow redundancy, so it cannot tolerate loss. Yu et al.’s scheme [27] adopts thumbnail-preserving secret sharing with (k,n) threshold, enabling recovery with *k* shadows even if n−k are lost. HCSS-GB and IBESS are built on (k,n) threshold mechanisms, where *k* or more shadows ensure lossless recovery, thus supporting loss tolerance.
- **Shadow Image Management:** This feature evaluates the efficiency of storing and organizing shadow images, focusing on reducing redundancy and facilitating large-scale management. Cheng et al.’s scheme [24] generates meaningful shadows by embedding shares into different carrier images, leading to high redundancy, as well as Yu et al.’s scheme [27]. Chowdhury et al.’s TPE scheme [31] preserves thumbnails in shadow images, allowing users to preview and manage via thumbnails, thus achieving efficient management. HCSS-GB and IBESS derive all shadows from blurred variants of a single carrier image, reducing redundancy via shared carrier characteristics and enabling efficient management.
- **Permission Hierarchy:** This feature refers to the ability to differentiate user access to secret information through tiered shadow quality, enabling fine-grained access control. Cheng et al.’s scheme [24] generates shadows with uniform visual quality, lacking privilege-based differentiation, thus no hierarchy, as well as Yu et al.’s scheme [27]. Chowdhury et al.’s TPE scheme [31] adjusts privacy via thumbnail block size (e.g., 16 × 16 to 64 × 64), where larger blocks reduce leakage risk for low-privilege users, supporting basic hierarchy. HCSS-GB and IBESS control shadow blur degrees via sigma, where high-privilege users receive less blurred shadows and low-privilege users receive more blurred ones, supporting explicit hierarchy.
- **Information Leakage Resistance:** This feature measures the ability to prevent unauthorized users from inferring the original secret from shadows, which is critical for privacy protection. Cheng et al.’s scheme [24] ensures shadows are visually similar to carriers but unrelated to the secret image, thus resisting leakage, as well as Yu et al.’s scheme [27]. Chowdhury et al.’s TPE scheme [31] retains thumbnails of the original image, which may leak semantic information (e.g., facial features), thus weak resistance. HCSS-GB and IBESS generate shadows via Gaussian blur and secret sharing, with no visual correlation to the secret image, thus resisting leakage.
- **Sharing Efficiency:** This feature is quantified by sharing time and computational overhead, critical for practical applications (especially real-time scenarios). The evaluation is based on 256 × 256 grayscale images under (7,8) threshold:
  **Low:** Sharing time > 100 s. Cheng et al.’s scheme [24] requires repeated selection of polynomial coefficients to match carrier high bits; HCSS-GB involves iterative checks of MSB matching.
  **Moderate:** Sharing time 10–100 s. Chowdhury et al.’s TPE scheme [31] avoids complex screening but involves chaotic sequence generation, being 17 times faster than traditional methods; Yu et al.’s scheme [27] optimizes screening via thumbnail constraints, reducing time.
  **High:** Sharing time < 10 s. IBESS uses bit expansion to avoid iterative checks, with (7,8) threshold taking 2.1 s.

## 7. Conclusions

To address the critical challenges in secret image sharing (SIS)—including unmanageable meaningless shadow images, inadequate fine-grained access control, and inefficient trade-offs between storage and speed in traditional schemes—this study proposes two novel solutions based on Shamir’s secret sharing: the HCSS-GB and the IBESS. These schemes are designed to fill key gaps in existing SIS research by prioritizing both practical manageability and scenario-specific efficiency.

HCSS-GB addresses shadow management and hierarchical access needs by generating meaningful shadows through gradient Gaussian blur, linking all shadows to a single carrier image to enable intuitive tiered control. In contrast, IBESS prioritizes sharing efficiency via a bit expansion mechanism, making it well-suited for real-time processing scenarios. Together, these two schemes form a dual-path solution that caters to diverse secure distributed image sharing requirements, balancing usability and performance across different application contexts.

The core contributions of this work lie in three interconnected advancements. It realizes meaningful shadow generation integrated with hierarchical access control, simplifying shadow management in real-world applications. It achieves an integrated solution through two complementary schemes: HCSS-GB focuses on storage optimization by maintaining a 1:1 storage ratio with the original image, while IBESS prioritizes speed enhancement to significantly accelerate processing in high-threshold scenarios. Additionally, it quantifies how carrier image attributes—specifically $C_{\sigma }$ and $S_{L}$—influence shadow distinguishability, providing actionable guidance for optimal carrier selection in hierarchical control design.

Experimental validation confirms that both schemes support lossless secret reconstruction, with perfect performance in metrics such as PSNR, MSE, and SSIM. HCSS-GB excels in scenarios requiring strict format consistency and hierarchical management, while IBESS stands out in time-critical tasks due to its high processing efficiency. Notably, carrier images with high $C_{\sigma }$ and $S_{L}$ values significantly enhance shadow distinguishability, directly improving the effectiveness of hierarchical access control.

Current research still has certain limitations: HCSS-GB exhibits suboptimal sharing speed under high thresholds, IBESS incurs doubled storage overhead, the current design does not explicitly address resistance to advanced noise attacks on shadow images, and formal specifications of access control policies remain underdeveloped. Future research will focus on optimizing HCSS-GB’s controlled sharing model to enhance sharing speed, reducing IBESS’s storage footprint through compression-friendly bit expansion strategies, strengthening shadow robustness against common image processing attacks such as cropping and noise addition, extending the hierarchical access mechanism to support multi-level authorization in large-scale distributed systems, and exploring the formal specification of access control policies to enhance the practicality and security of our scheme for real-world applications.

## Figures and Tables

**Table 1:** List of Symbols and Definitions.

Symbol	Definition
$Z_{p}$	Prime field with *p* elements, where *p* is a prime number.
*k*	Threshold parameter for secret reconstruction, indicating the minimum number of shares required to recover the secret.
$X={\left\{x_{i}\right\}}_{i=1}^{n}$	Set of participant identifiers, where each $x_{i}$ is an element of $Z_{p}\setminus \left\{0\right\}$.
$d=(d_{0},\ldots ,d_{k-1})$	Exponent tuple used for polynomial construction, where each $d_{j}$ is an exponent in the polynomial.
$a=(a_{0},\ldots ,a_{k-1})$	Coefficient vector for the polynomial, with $a_{0}$ representing the secret *S*.
$V\in Z_{p}^{n\times k}$	Generalized Vandermonde matrix, where each element $V_{ij}$ is computed as $x_{i}^{d_{j}}\;\mathrm{mod}\;p$.
$s=(s_{1},\ldots ,s_{n})$	Share vector, where each share $s_{i}$ is computed as $\sum_{j=0}^{k-1}a_{j}x_{i}^{d_{j}}\;\mathrm{mod}\;p$.
P(i,j)	Pixel value at (i,j) in the plaintext image P.
C(i,j)	Pixel value at (i,j) in the carrier image C.
Ω(P(i,j),BC(i,j))	Matching highest bits count between pixel P(i,j) in plaintext and BC(i,j) in blurred carrier image, ranging from 1 to 8.
PSC	Preview Shadow Image, a visually similar approximation of the final shadow image used for quick evaluation.
BC	Blurred Carrier Image, a version of the carrier image C with different levels of Gaussian blur applied.
SC	Shadow Image used for reconstructing the original secret image.
GaussianBlur(I,σ,K)	Gaussian blurring function applied to image *I*, where the kernel size is fixed as K×K and σ is the standard deviation.

**Table 2:** PSNR, MSE and SSIM values of the images recovered by HCSS-GB and IBESS.

(k,n)-Threshold	(2,2)	(3,3)	(3,4)	(4,5)	(5,6)	(6,7)	(7,8)
HCSS-GB	*∞*/0/1	*∞*/0/1	*∞*/0/1	*∞*/0/1	*∞*/0/1	*∞*/0/1	*∞*/0/1
IBESS	*∞*/0/1	*∞*/0/1	*∞*/0/1	*∞*/0/1	*∞*/0/1	*∞*/0/1	*∞*/0/1

**Table 3:** $C_{\sigma }$ and $S_{L}$ of experimental images with all sizes being 256×256.

Image Name	$C_{\sigma }$	$S_{L}$
cameraman.bmp	62.34	2241.4243
brain.bmp	61.51	678.6579
peppers.bmp	53.26	681.4585
barbara.bmp	52.6	1450.5473
plane.bmp	45.61	1151.2901
watch.bmp	34.14	750.4396
baboon.bmp	32.71	2161.7273

**Table 4:** Comparison of storage space for shadow images of HCSS-GB and IBESS.

(k,n)-Threshold	(2,2)	(3,3)	(3,4)	(4,5)	(5,6)	(6,7)	(7,8)
HCSS-GB	1	1	1	1	1	1	1
IBESS	≈2	≈2	≈2	≈2	≈2	≈2	≈2

**Table 5:** Comparison of key characteristics among different schemes.

Scheme	Loss Tolerance	Shadow Image Management	Permission Hierarchy	Information Leakage Resistance	Sharing Efficiency
Cheng et al. [24]	Yes	No	No	Yes	Low
Chowdhury et al. [31]	No	Yes	Yes	No	Moderate
Yu et al. [27]	Yes	No	No	Yes	Moderate
HCSS-GB	Yes	Yes	Yes	Yes	Low
IBESS	Yes	Yes	Yes	Yes	High

## Data Availability

The original contributions presented in this study are included in the article. Further inquiries can be directed to the corresponding authors.
